# Reactive oxygen species in haematopoiesis: leukaemic cells take a walk on the wild side

**DOI:** 10.1186/s13046-018-0797-0

**Published:** 2018-06-26

**Authors:** Rodrigo Prieto-Bermejo, Marta Romo-González, Alejandro Pérez-Fernández, Carla Ijurko, Ángel Hernández-Hernández

**Affiliations:** 1 Department of Biochemistry and Molecular Biology, University of Salamanca, Lab. 122, Edificio Departamental, Plaza Doctores de la Reina s/n, 37007 Salamanca, Spain; 2 IBSAL (Instituto de investigación Biomédica de Salamanca), Salamanca, Spain

**Keywords:** Reactive oxygen species (ROS), Redox signalling, Oxidative stress, Haematopoietic stem cells (HSCs), Leukaemia, NADPH oxidase (Nox), Mitochondria

## Abstract

Oxidative stress is related to ageing and degenerative diseases, including cancer. However, a moderate amount of reactive oxygen species (ROS) is required for the regulation of cellular signalling and gene expression. A low level of ROS is important for maintaining quiescence and the differentiation potential of haematopoietic stem cells (HSCs), whereas the level of ROS increases during haematopoietic differentiation; thus, suggesting the importance of redox signalling in haematopoiesis. Here, we will analyse the importance of ROS for haematopoiesis and include evidence showing that cells from leukaemia patients live under oxidative stress. The potential sources of ROS will be described. Finally, the level of oxidative stress in leukaemic cells can also be harnessed for therapeutic purposes. In this regard, the reliance of front-line anti-leukaemia chemotherapeutics on increased levels of ROS for their mechanism of action, as well as the active search for novel compounds that modulate the redox state of leukaemic cells, will be analysed.

## Background

The emergence of oxygen in the Earth’s atmosphere, more than 2 billion years ago, was a key event for life on this planet. The access to oxygen allowed eukaryotic cells to adopt a more efficient metabolism, which sped up the evolution of eukaryotes. However, aerobic metabolism also has a dark side: the production of reactive oxygen species (ROS) by the partial reduction of oxygen. Some representative examples are superoxide (O_2_^−^), hydrogen peroxide (H_2_O_2_) and hydroxyl radical (OH^−^) [1]. ROS have traditionally been considered toxic by-products of cellular respiration. They are highly reactive and are able to modify all kinds of biomolecules in the cell. For this reason, oxidative stress has been related to ageing [2] and pathological conditions such as cancer [3]. However, during the last decades it has been determined that a moderate production of ROS may be required for the control of cellular signalling and gene expression [4]. The term *redox signalling* is increasingly used, and should be distinguished from the concept of *oxidative stress*.

Quantitatively, the mitochondrion is the most important source of ROS in the cell. Some enzymatic systems also contributing to ROS production are xanthine oxidoreductase (XOR), uncoupled NO synthase (NOS), cyclo-oxygenase (Cox), cytochrome P450 mono-oxygenase, myeloperoxidase (MPO), lipoxygenase and NADPH oxidases (Nox). Among these cellular systems, the NADPH oxidase family has exclusively evolved to produce ROS as a primary function [5]. Curiously, the first member of the family, phagocyte oxidase, was identified in the haematopoietic system. This enzyme produces large amounts of ROS which are required for pathogen elimination [5]. Genetic disorders associated with this oxidase cause chronic granulomatous disease (CGD), an innate immunity defect characterised by recurrent fungal and bacterial infections [6]. In addition to Nox2, the catalytic subunit of the phagocyte oxidase, there are 6 other family members (Nox1, Nox3, Nox4, Nox5, Duox1 and Duox2). Duox1 and 2 present a peroxidase-like domain and together with Nox5 could be activated by calcium [5].

The production of intracellular ROS in response to growth factors was suggested almost 40 years ago [7]. It was later observed that low concentrations of H_2_O_2_ induce cell growth [8]. Nowadays, there is a plethora of evidence showing the importance of ROS production for cellular signalling in response to growth factors and other signals [9]. A proportion of ROS is catalytically generated and eliminated in the cell in response to extracellular signals. The fact that OH^-^ removes electrons from surrounding biomolecules in an unspecific manner and O_2_^−^ signalling function requires its transformation into H_2_O_2_ suggests that H_2_O_2_ most likely plays a role as a true second messenger [10]. On the basis of this hypothesis, it would be interesting to elucidate how specificity is achieved in the context of redox signalling. It is, however, tempting to speculate about the formation of a ROS gradient from the source of production, where proteins under the influence of such a gradient would be oxidised, while others out of reach would be immune to increases in the level of ROS.

**Fig. 1:**
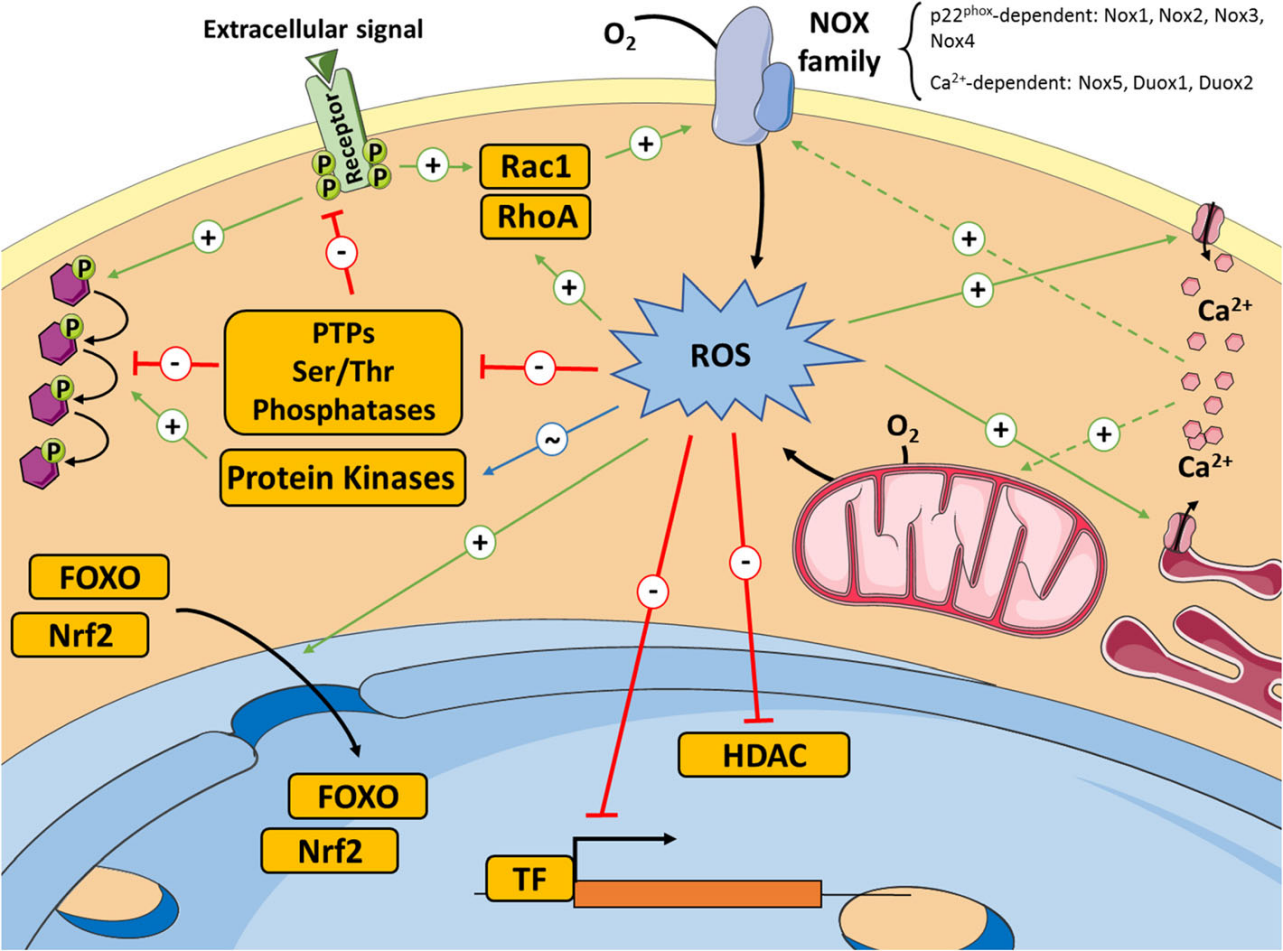
General landscape of redox singnalling. Here we present the main molecular targets of ROS described to date: classical signalling proteins, such as kinases, phosphatases or small GTPases; transcription factors and epigenetic modullators; and calcium fluxes, with which there exists a complex cross-talk (see text). Red arrows mean inhibition of the target or process; green arrows mean activation; blue arrow with ‘~’ symbol means that both activation and inhibition can be achieved depending on the specific target. Dotted lines indicate propose or not empirically demonstrated relationship

To fully comprehend the concept of *redox signalling*, it is necessary to understand how ROS influence cellular signalling and gene expression (Fig. 1). Compelling evidence suggests that the reversible oxidation of specific amino acids, such as certain cysteine residues, regulates protein function [10]. Modification of proteins with reactive cysteines is thought to be a general mechanism to mediate redox signalling. One of the best known examples is the family of the protein tyrosine phosphatases (PTPs). All these proteins are characterised by the presence of a conserved cysteine residue required for catalysis [11], which makes them highly susceptible to oxidation [12]. Reversible oxidation of PTPs has been suggested as an important mechanism in the regulation of the activity of these enzymes in vivo [13].

The list of signalling proteins regulated through oxidation is continuously growing. Serine/threonine phosphatases can be inhibited [14], kinases are subject of either activation or inhibition [15] and the activity of Rac1 [16] and RhoA [17] GTPases is reported to be increased when key amino acids are oxidized.

There is also a complex cross-talk between ROS and Ca^2+^ levels. Calcium channels seem to be regulated by redox mechanisms in different subcellular locations and, concomitantly, ROS production by NADPH oxidases and mitochondria may be induced by calcium [18].

The list of transcription factors, such as AP-1, p53, NF-κB, or HIF1α, whose DNA binding affinity can be inhibited by oxidation, is also growing. The AP endonuclease 1/redox factor 1 (APE1/Ref-1) complex, involved in base excision repair, keeps transcription factors in a reduced active state [19]. ROS have also been shown to regulate the accumulation of transcription factors in the nucleus, such as the Forkhead box protein O 4 (FOXO4) [20] and nuclear factor erythroid 2-related factor 2 (Nrf2) [21]. Interestingly, FOXOs and Nrf2 participate in cellular response to oxidative stress, since they have the ability to bind ARE (antioxidant response elements) and activate the expression of antioxidant and detoxifying enzymes. The activity of epigenetic modifiers, such as some histone deacetylases (HDACs), can also be inhibited by oxidation [22].

## Redox signalling in haematopoiesis

Haematopoiesis constitutes one of the paradigms of cell differentiation, since a single cell type, the haematopoietic stem cell (HSC), gives rise to all mature blood lineages. Unlike other differentiation processes restricted to embryonic development, blood cell production continues to occur throughout the entire life cycle. To ensure correct haematopoiesis, the balance between differentiation and self-renewal in HSCs must be strictly regulated [23]. Moreover, the use of HSCs in regenerative medicine is noteworthy, and a better understanding of HSCs biology could contribute to improving their usefulness.

### The niche contributes to the maintenance of a low level of ROS in HSCs

Compelling evidence suggests that ROS are important to regulate the balance between self-renewal and differentiation of adult stem cells. A low level of ROS would contribute to maintaining the pluripotency of these cells, whereas a higher amount would commit them to a restricted lineage [24]. In adults, HSCs reside and differentiate in bone marrow (BM), where different cell types, soluble factors and anatomical structures collaborate to maintain their characteristics. This complex environment is generally known as the *niche* [25], where different regions have been distinguished: the endosteal niche, defined by the osteoblasts; the vascular niche, composed by BM sinusoidal endothelial cells (BMSECs); and the perivascular niche, where CXC chemokine ligand 12 (CXCL-12)-abundant reticular cells (CAR cells) and Nestin^+^ mesenchymal stem cells are present [26]. Apart from comprising different cell types, a fundamental difference among these niches is access to oxygen, which should be more readily available within the vascular and perivascular niche than in the endosteal niche.

The accepted idea is that most quiescent HSCs remain under hypoxic conditions in BM [24]. A more restricted access to oxygen would result in lower ROS content, which could have relevant functional consequences. A seminal contribution by Jang and Sharkis showed that a high level of ROS is detrimental for HSCs function [27]. They characterized two different HSC populations according to the intracellular levels of ROS. The ROS^low^ population showed greater quiescence and self-renewal potential, while in the ROS^high^ population the haematopoietic reconstitution capacity was hampered. They also suggested that the ROS^low^ population is located within the endosteal niche, where cells have less oxygen availability and therefore lower levels of intracellular ROS. This situation would promote their quiescence and maintain their reconstitution capacity. In addition to their location, some work has highlighted the relevance of niche cells in the maintenance of a reduced ROS concentration in HSCs through a transference of ROS from these cells to BM stromal cells [28].

HSCs receive multiple stimuli from the surrounding niche that influence their ability to remain quiescent, undergo self-renewal or differentiate. One of the most important signals is the stromal cell-derived factor-1 (SDF-1, also named CXCL12) which binds to the CXCR4 receptor in HSCs. CXCL12 belongs to a large family of chemoattractive cytokines that act through G-protein-coupled receptors. This cytokine is produced by CAR cells in the bone marrow niche, and was originally described as being involved in the proliferation of B cell precursors. Later on its essential role for HSCs homing was discovered [29]. The CXCL12/CXCR4 axis regulates important processes such as homing, quiescence/proliferation or migration in these cells. Interestingly, protection against oxidative stress has recently emerged as an important mechanism of CXCL12/CXCR4 signalling in the maintenance of HSCs homeostasis [30]. As recently reviewed, the alteration of this signalling pathway may contribute to leukaemogenesis [31]. In addition to its involvement in haematopoiesis, CXCL12/CXCR4 signalling is required for stem cell migration and homing in other developmental processes, such cardiogenesis, angiogenesis and neurogenesis [29], and also for cancer cell migration and metastasis [32].

### Intrinsic factors that control ROS levels in HSCs

Several reports suggest that the most primitive HSCs, those with the capacity for long-term reconstitution (LT-HSCs), are located at the endosteal niche, where they can face hypoxic conditions [33]. The lack of oxygen requires for them to adopt an anaerobic metabolism, which is linked to a decrease in ROS production [24]. However, there are some reports suggesting that the reduced ROS content in HSCs is independent of their anatomical location [34]. Therefore, besides localization within BM niches, there must be intrinsic factors that contribute to maintaining the low levels of ROS detected in HSCs. As will be discussed later on, some of these factors have been revealed through gene-targeting experiments in mice, where HSCs are shown to have an increased level of ROS and an impaired functionality.

Hypoxia inducible factor 1 (HIF-1) is a transcription factor essential for the adaptation to low O_2_ pressure. HIF-1 is a heterodimeric protein that consists of a constitutively expressed subunit (HIF-1β) and an inducible subunit (HIF-1α) stabilised upon hypoxic conditions. Under normoxia, HIF-1α hydroxylation targets the protein for degradation [35]. Stabilization of HIF1α in HSCs seems to be important for maintaining the correct level of ROS. It has been described how the lack of HIF1α in HSCs leads to increased ROS production, which is related to overproliferation, engraftment defects, loss of stemness and senescence [36]. The expression of HIF1α can promote anaerobic metabolism instead of mitochondrial oxidative phosphorylation in HSCs [37]. This fact, together with reduced mitochondrial activity [24], would lead to the limited production of ROS in these cells.

There is increasing evidence showing that the Forkhead O (FoxO) subfamily of transcription factors plays an important role in the preservation of a low level of ROS in HSCs, which protects them from oxidative stress. The deletion of *FoxO1*, *FoxO3* and *FoxO4* in the hematopoietic system leads to a dramatic decrease in the number of HSCs, an increase of their cycling, and the expansion of myeloid lineage cells. Moreover, their reconstitution capacity is severely disrupted. These effects correlate with a significant increase in ROS in the HSCs compartment; therefore, antioxidant treatment could rescue the phenotype [38]. In agreement with these findings, the deletion of *FoxOs* is reported to reduce HSCs quiescence and to impair their self-renewal capacity as a consequence of the ROS-dependent activation of the p38 mitogen-activated protein kinase (MAPK) cascade [39].

The protein encoded by the *ataxia–telangiectasia mutated gene* (*Atm*) coordinates the cellular response to protect genomic stability against damage such as genotoxic and oxidative stress or DNA double strand breaks. Mutant variants of this gene cause ataxia telangiectasia [40], a disease characterised by severe neurological defects, and increased risk of developing T-and B-cell lymphomas and leukaemias [41]. There is evidence suggesting that *Atm* gene plays a role in maintaining a low level of ROS in HSCs. *Atm* loss correlates with a ROS increase in HSCs and the activation of the p38 MAPK cascade, which reduces the lifespan and the quiescence of HSCs. Interestingly, treatment with antioxidants or p38 inhibitors alleviates these effects [42].

Previous work has shown that p53 stabilization is required to avoid the exhaustion of HSCs upon oxidative stress [43]. However, this is a complex situation, since p53 accumulation can also lead to an increase in ROS; thus, initiating a vicious cycle that induces HSCs cell cycle arrest, senescence and eventually cell death [44].

Polycomb group (PcG) proteins are implicated in transcriptional repression through histone modifications, and are known to control the self-renewal of stem cells [45]. Deletion of one member of this family, *Bmi1*, induces premature HSCs senescence and deficient self-renewal [46]. These effects could be related to an increase in ROS. Also, it should be noted that Bmi1 can regulate mitochondrial ROS production [47].

Furthermore, the overall ROS levels in cells are a result of the balance between their generation and elimination. Although more work is still required to further understand the relevance of this relationship, it could be assumed that the activity of the cellular antioxidant systems is necessary for maintaining the low levels of ROS observed in HSCs [48].

### Redox signalling and the haematopoietic differentiation

HSCs are influenced by numerous cytokines, growth factors and other soluble factors that instruct their self-renewal, proliferation, differentiation or migration within the different niches. It has long been known that haematopoietic cytokine signalling is accompanied by ROS formation [49]. It has also been described that the levels of ROS in myeloid progenitors are higher than in HSCs [38], and that an increase in ROS triggers myeloid differentiation in animal models [50]. Some results have shown the importance of a moderate increase in ROS for the megakaryocytic differentiation of human HSCs [51]. More recently, a progressive increase in ROS during HSCs differentiation has been described [52]. Bearing the aforementioned in mind, the importance of redox signalling for haematopoiesis is unquestionable. Thus, the signalling pathways regulating haematopoiesis susceptible to redox regulation should be delineated. In the following paragraphs the possibilities regarding this association will be discussed.

An increase of ROS during haematopoietic cytokine signalling is necessary for the complete activation of the pathways that lead to differentiation, including AKT [51]. The phosphoinositide 3-kinase (PI3K)/AKT/mTOR (mammalian target of rapamycin) pathway is a good example illustrating that an appropriate level of ROS is essential for haematopoiesis. Simultaneous deletion of *AKT1* and *AKT2* greatly increases the quiescence of HSCs to the point of impairing their long-term reconstitution capacity [53]. This failed haematopoiesis correlates with a significant decrease in the ROS content of HSCs. However, the application of exogenous ROS could rescue the differentiation capacity of *AKT1* and *AKT2* deficient HSCs in vitro. On the contrary, constitutive AKT activation increases HSCs cell cycling and leads to leukaemogenesis [54]. Therefore, a proper supply of redox signalling is important for the appropriate activation of AKT, which in turn is involved in maintaining the correct level of ROS for HSCs differentiation.

The deletion of *tuberous sclerosis complex* (*TSC*) gene product TSC1 suggests that mTOR inactivation represses mitochondrial biogenesis, which would maintain low levels of ROS in the HSCs compartment [55].

The MAPK pathways are involved in the transmission of a great variety of extracellular signals, including those regulating haematopoiesis [56]. MAPK activation can sometimes be dependent on ROS production [57]. A sustained activation of the MEK/ERK pathway by thrombopoietin (TPO) is observed during megakaryocytic differentiation [58]. The complete activation of the MEK/ERK pathway depends on a moderate increase in ROS production [51]. As mentioned above, the p38 MAPK pathway can be aberrantly activated by oxidative stress, thus impairing HSCs function and engraftment [42, 59].

Given the susceptibility of PTPs to oxidation, these enzymes could be involved in the redox control of haematopoiesis. As recently shown, SHP1 oxidation is required for T cell development [60]. Gain-of-function mutations in the *PTPN11* gene, encoding SHP2, lead to Noonan syndrome, juvenile myelomonocytic leukaemia (JMML), myelodysplastic syndrome, B cell ALL, and AML. Some of these mutations have been shown to induce an increase in cellular ROS, which could alter the redox signalling balance and be related to the pathogenicity [61].

Canonical Wnt signalling promotes β-catenin stabilisation, and has been related to the control of HSCs homeostasis [62]. β-catenin levels are important for regulating HSCs quiescence and the interaction with the BM niche [63]. It has been shown that Wnt/β-catenin can be under redox regulation by nucleoredoxin [64]. Moreover, β-catenin levels are controlled by the protein tyrosine phosphatase PTPN13 [65]. Taking all these facts into consideration, it is tempting to speculate that the Wnt/β-catenin signalling could be a target of ROS during haematopoiesis.

## Oxidative stress and redox signalling in leukaemia

Since leukaemogenesis is a multistep process, it is difficult to identify a single driving force. Mutations affecting oncogenes, DNA repair genes and tumour suppressor genes may be some of the causes of leukaemogenesis. Such mutations would confer a proliferative advantage to leukaemic cells and a way to avoid differentiation [66]. With the discovery of oncogenes, a great deal of cancer research has been focused on their alterations, disregarding other cellular processes such as the metabolic changes that occur during tumour transformation. Nowadays, the implication of the metabolic reprogramming exhibited by leukaemic cells is receiving much attention [67]. It has long been known that increased production in ROS is one of the defining characteristics of tumour cells [68], and leukaemic cells are no exception, as they also exhibit elevated level of ROS. This feature has been observed in numerous leukaemic cell lines and also in the cells from patients with different types of leukaemia, such as chronic myeloid leukaemia (CML) [69, 70], acute myeloid leukaemia (AML) [71], T-cell acute lymphoblastic leukaemia (T-ALL) [72], acute lymphocytic leukaemia (ALL) [73], chronic lymphocytic leukaemia (CLL) [74], and in myeloproliferative neoplasms (MPNs) [75]. Therefore, it is generally accepted that increased ROS production is important for the progression of haematological malignancies [76, 77].

In this section some of the conditions that might explain the situation of oxidative stress with respect to leukaemic cells will be analysed. Moreover, we will delve into the relationship between leukaemia-related oncogenes and oxidative stress.

### Sources of ROS in leukaemia

Haematopoietic cytokine signalling occurs in parallel with an increase in ROS production [49]. It has been shown that TPO elicits ROS production in human HSCs, an event required for their differentiation [51]. The results of other laboratories are in agreement with this finding, thus highlighting the relevance of NADPH oxidases for megakaryopoiesis [78]. Recent reports suggest the implication of NADPH oxidases in the differentiation of other haematopoietic lineages such as macrophages [79] or granulocytes [80]. Different Nox isoforms are found in haematopoietic cells with expression patterns that may vary among the different lineages [81]. Taken together, these data support the idea that NADPH oxidase-driven ROS production is important for haematopoiesis.

Compelling evidence also illustrates the importance of NADPH oxidases as a source of ROS in leukaemia. Their enzymatic activity appears to be required for cell proliferation in AML patients [71]. The presence of a truncated form of the granulocyte colony-stimulating factor (G-CSF) receptor is associated with a high risk of developing AML. Interestingly, this defective receptor induces an increase in ROS through NADPH oxidases, which could be assumed to be related to disease progression [82]. It seems that Nox2 activity could account for the immunoresistance of chronic myelomonocytic leukaemia (CMML) [83] and CML cells [84]. There are two different reports that link Nox5 activity with two types of lymphoid leukaemia. Kamiguti et al. suggest that Nox5-generated ROS contribute to the activation of malignant B cells in hairy cell leukaemia through the inactivation of SHP1 [85]. In addition, another report supports the requirement of Nox5 for cell transformation driven by the human T-cell leukaemia virus type 1 (HTLV-1), which is associated with the development of adult T-cell leukaemia (ATL) [86].

Although HSCs exhibit low mitochondrial metabolism, recent work suggests the importance of mitochondrial function for their survival [87] and self-renewal [88]. Mitochondrial biogenesis is activated as HSCs differentiate [89]. Additionally, their metabolism shift towards oxidative phosphorylation, thus leading to increased ROS production, which may drive their entry into the cell cycle [90, 91]. An interesting question to be addressed in the future is whether mitochondrial ROS regulate cellular signalling during haematopoiesis. A recent report showing that mitochondrial ROS downregulate Notch signalling, an event required for HSCs differentiation [52], supports this notion.

Almost a century ago, Otto Warburg demonstrated that tumour cells display important differences with respect to their healthy counterparts in terms of the use of glucose. Even in aerobic conditions, cancer cells produce high amounts of lactic acid from glucose. In other words, they rely on glucose fermentation as a way of obtaining energy, a phenomenon known as “Warburg effect” [92]. Despite this metabolic adaptation, mitochondria remain functional and tumour cells can still carry out an oxidative phosphorylation for energy production [93]. In fact, it is accepted that mitochondrial respiration contributes to tumourigenesis [94]. Some authors have identified mitochondria as the main source of ROS in leukaemia. A recent report suggests enhanced mitochondrial ROS production in CML patients [70]. Moreover, Jitschin et al. have demonstrated that increased mitochondrial metabolism and biogenesis correlate with elevated ROS levels in CLL patients [95].

Mitochondrial DNA (mtDNA) is not packed as chromatin, and therefore is more susceptible to oxidative damage than nuclear DNA. In addition, its lack of introns increases the probability of mutations occurring within coding regions. As a result, an increased mitochondrial ROS production can jeopardise the stability of mtDNA. Concomitantly, oxidative stress could promote DNA damage and mutation, which would lead to a further increase in ROS production that would eventually promote leukaemogenesis [96]. In fact, mtDNA alterations are thought to be a risk factor for developing leukaemia, drug resistances, and disease progression. Mutations and alterations in mtDNA have been reported in the four main types of leukaemia: CML [97, 98], AML [99], CLL [100], and ALL [101]. More studies are needed to determine the precise contribution of mitochondria, and their alteration, to the oxidative stress *status* in leukaemia, and to dissect the factors that increase mitochondrial ROS production. However, the evidence accumulated so far supports the importance of such contribution.

As will be discussed below, there are other potential sources of ROS in leukaemia. Although the implication of ROS in this process has not yet been proven, they have been associated with haematological malignancies. This raises the possibility that other sources of ROS may also contribute to oxidative stress in leukaemia.

Xanthine oxidoreductase (XOD) is involved in purine catabolism and produces ROS as by-products. A high XOD expression has been shown to correlate with high-grade malignancy in solid tumours [102]. The implication of XOD in leukaemia has not formally been addressed. However, an increase of XOD activity in AML patients has been reported [103, 104]. It is tempting to speculate that this enzyme may also contribute to oxidative stress in leukaemia.

Lipoxygenases and cyclooxygenases participate in arachidonate metabolism for the production of eicosanoid mediators. Two different studies from the same group have recently shown the relevance of arachidonate 5-lipoxygenase (*Alox5*) [105] and arachidonate 15-lipoxygenase (*Alox15*) [106] genes for the survival of CML leukaemia stem cells (LSC). The expression of both genes is upregulated by BCR-ABL and is required for the development of the disease in vivo. Although these studies do not address whether lipoxygenases-derived ROS are required for disease onset, one could speculate about such a possibility. Overexpression of cyclooxygenase-2 (Cox-2), an enzyme also involved in arachidonate metabolism, has been linked to different haematological malignancies and is considered to be a poor prognostic factor [107].

Cytochrome P-450 enzymes (*CYP*) have an important role as a detoxifying system. Certain *CYP* gene polymorphisms have been associated with a higher risk of developing different kinds of solid tumours [108]. In addition, polymorphisms affecting *CYP* and other genes encoding potential ROS sources, such as myeloperoxidase (*MPO*) and NAD(P)H: quinone oxidoreductase 1 (*NQO1*), have been related to a predisposition to develop ALL [109].

### Antioxidant defences in leukaemic cells

As previously noted, the redox homeostasis depends on the balance between ROS producers and cellular antioxidant systems. A defective antioxidant defence could account for the increased levels of ROS detected in leukaemic cells. Indeed, this fact has been observed in both myeloid and lymphoid leukaemias. The activity of several antioxidant enzymes was reduced in CML patients [110]. Furthermore, the presence of an impaired antioxidant defence system has recently been reported in AML patients [111]. The level of antioxidant enzymes, such as glutathione peroxidase, catalase and superoxide dismutase, is reduced in ALL patients [74]. Moreover, a decrease in the activity of catalase and superoxide dismutase, together with a lower level of reduced glutathione, has been detected in CLL samples [112].

Therefore, a diminished cellular antioxidant capacity may explain the oxidative stress found in leukaemic cells; however, this is not a general statement. In fact, the antioxidant capacity is often increased in leukaemia patient cells [113]. Depending on the scenario, certain antioxidant enzyme levels could be increased or decreased. The down-modulation of some antioxidant systems would contribute to the high level of ROS found in these cells, while the upregulation of other systems would allow the cells to survive under a situation of permanent oxidative stress without surpassing a deadly threshold. In other words, modulation of antioxidant defences allow leukaemic cells to increase their redox signalling without setting in motion an irreparable state of oxidative stress [113].

### Leukaemic oncogenes induce ROS production

Genetic alterations caused by chromosomal translocations or activating mutations are observed in several leukaemia-related oncogenes. Recent evidence shows that some of these changes are able to induce ROS production, which could be an important driving force for leukaemogenesis.

*BCR-ABL* is probably one of the best examples of leukaemia-driving oncogenes; the expression of the chimeric BCR-ABL tyrosine kinase is sufficient for cell transformation in CML [114]. Several reports support the idea that BCR-ABL is able to induce ROS production from different sources, including mitochondria [115], the upregulation of NADPH oxidase activity [116] by increasing p47^Phox^ expression [117] and an enhanced glucose metabolism [118]. It seems that the increased ROS production would be important for cell growth and transformation. The oxidative stress would increase genomic instability and mutagenesis [119]. This vicious circle of oxidative stress-DNA mutation could affect the *BCR-ABL* gene itself, causing the occurrence of BCR-ABL protein variants resistant to the standard tyrosine kinase inhibitors (TKi) used in clinical practice [120]. About 30% of AML patients exhibit internal tandem duplications in the FMS-like tyrosine kinase-3 (FLT3) receptor, giving rise to the FLT3/ITD oncokinase [121]. FLT3/ITD induces ROS production through NADPH oxidases [122], which has been suggested to contribute to leukaemic transformation [121, 123]. Activating mutations in either *NRAS* or *KRAS* genes occur in AML, juvenile myelomonocytic leukaemia (JMML), chronic myelomonocytic leukaemia (CMML) [124], childhood ALL [125] and myelodysplastic syndromes (MDS) [126]. It has long been known that cell transformation by *RAS* relies on NADPH oxidase-driven ROS production [127], which induces growth factor-independent proliferation of human CD34^+^ cells [128]. Thus, it has been proposed that Ras-dependent ROS production could contribute to leukaemogenesis [126].

The activating mutation of JAK2^V617F^ often appears in MPNs [129]. Recent reports detected an increased ROS production in MPNs carrying the JAK2^V617F^ mutation, as a consequence of NADPH oxidase activation [75] or catalase downregulation [130]. Experimental evidence supports the hypothesis that increased ROS production in the HSC compartment would contribute to MPN progression [130].

All the examples of oncogenes analysed so far encode proteins that activate signal transduction pathways driven by protein phosphorylation. It is intriguing that in all these cases the activation of NADPH oxidases contributes to increased ROS production. Although there are other mechanisms through which these oncogenes may enhance ROS generation, it is reasonable to consider the activation of NADPH oxidases as the most likely cause. Thus, oxidation-mediated PTP inactivation and subsequent boosting of phosphorylation cascades driven by oncogenes through this mechanism could be proposed.

The *FIP1L1-PDGFRA* fusion gene, which leads to the constitutive activation of PDGF receptor, is the most frequent genetic defect in hypereosinophilic syndrome (HES), and has been found in approximately 50% of chronic eosinophilic leukaemia (CEL) patients. The expression of this kinase has been associated with the down-regulation of different antioxidant enzymes; thus, suggesting an increase in ROS could be a possible oncogenic factor in this kind of leukaemia [131].

Mutations of the *Isocitrate dehydrogenase 1 (IDH1)* and 2 (*IDH2*) genes have been recently found to be common in AML [132]. Most of them are gain-of-function mutations. Instead of oxidising isocitrate to α-ketoglutarate (α-KG), rendering NADPH, the mutant enzymes reduce α-KG to 2-hydroxyglutarate (2-HG) with the consumption of NADPH [133]. These mutations alter redox homeostasis, first by diminishing cellular NADPH content, and then because 2-HG acts as an oncometabolite that contributes to leukaemia transformation by increasing oxidative stress [134].

## The use of ROS as a therapeutic target in haematological malignancies

**Fig. 2:**
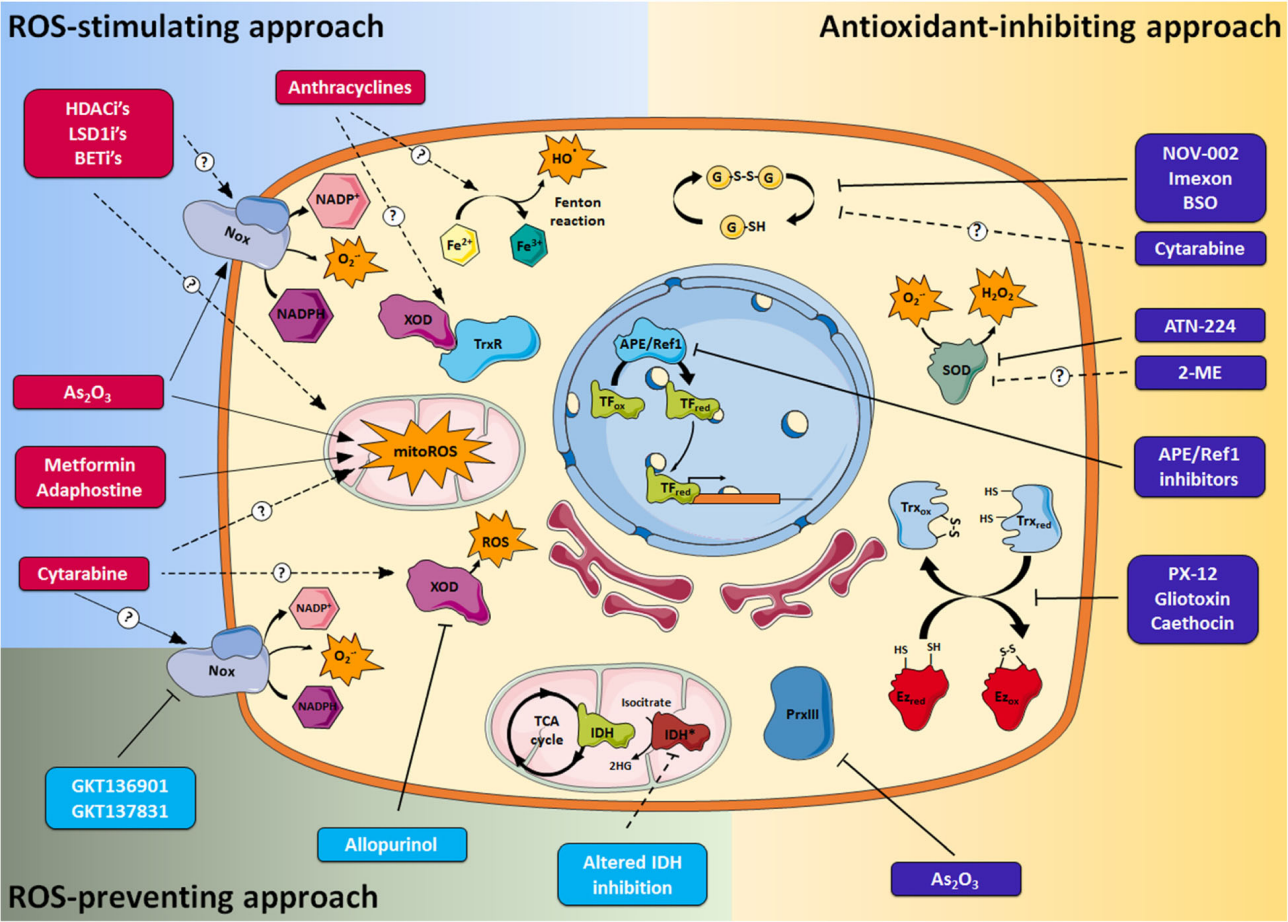
Redox-based treatment of haematological malignancies. The diagram shows three different approaches relying on ROS modulation of leukaemic cells: increasing ROS either upon stimulation of their cellular sources or inhibition of the antioxidant systems, in order to exceed the amount compatible with life, and decreasing the ROS, mainly by inhibition of cellular sources, thereby depleting the proliferative advantage that these products provide to the cancer cell. Some examples of drugs or plausible therapeutic strategies and their cognate cellular targets illustrate the three approaches described (see text for details). Dotted arrows with question marks indicate plausible but not completely established mechanisms of action

The permanent condition of the oxidative stress of leukaemic cells may offer interesting therapeutic options, and as a result the possibility of developing redox chemotherapeutics has received much more attention in recent years [135, 136]. In this regard, increasing or decreasing ROS levels may provide a suitable global strategy against leukaemia (Fig. 2 and Table 1). Theoretically, a pro-oxidant treatment could lead to an unbearable situation of oxidative stress incompatible with the viability of leukaemic cells. Conversely, the reduction of ROS levels could also be a feasible option due to the importance of ROS in sustaining leukaemic cell proliferation and survival. The lower levels of ROS detected in healthy cells could offer a certain type of specificity that would allow these cells to survive or become less affected.

**Table 1:** Chemotherapeutics that alter cellular redox balance

Compound	Status	ROS modulation	Clinical Trials
Arsenic trioxide (As_2_O_3,_ trisenox)	FDA-approved for the treatment of **relapsing AML.**	**↑** ROS: Inhibits MRC, TrxR and SOD; induces the expression of Nox2 complex genes and depletes Prx III	**Phase II** study of As_2_O_3_ plus decitabine and cytarabine AML with p53 mutations (NCT03381781, not yet recruiting). **Phase I** studies of As_2_O_3_ plus decitabine (NCT00671697, completed in 2011), plus cytarabine and idarubicin (NCT00093483, completed in 2009) for AML treatment. **Phase II/III** study of As_2_O_3_ plus altezumab and other chemoteraputic agents for AML or MDS treatment (NCT00454480, completed).
Metformin	FDA-approved for the treatment of **type 2 diabetes.**	**↑** ROS: Inhibits mitochondrial ATP production	**Phase II** study for CLL treatment (NCT01750567, recruiting). **Phase I** study of metformin plus ritonavir for relapsed/refractory MM or CLL treatment (NCT02948283, active). **Phase I** study for relapsed childhood ALL (NCT01324180; completed). **Phase I** study of metformin plus cytarabine for relapsed/refractory AML treatment (NCT01849276, active).
Tigecycline	FDA-approved for **complicated skin and intra-abdominal infections**.	**↑** ROS: Inhibits mitochondrial biogenesis	**Phase I** study for AML treatment (NCT01332786, completed in 2015). In vitro study of tigecycline in cells from CML patients (NCT02883036, not yet recruiting).
NOV-002	Under clinical trial.	**↑** ROS: GSSG mimetic	**Phase III** study of NOV-002 plus cis-platinum and placlitaxel for NSCLC treatment (NCT00347412, completed in 2010).
2-methoxyestradiol (2-ME, panzem)	FDA-approved for **ovarian cancer**, **metastatic malignant melanoma**, **MM** and **pancreatic adenocarcinoma**.	**↑** ROS: SOD inhibitor	**Phase II** study for the treatment of relapse or plateau phase myeloma (NCT00592579, completed in 2008).
Imexon	FDA-approved for **pulmonary arterial hypertension**, **ovarian cancer**, **multiforme glioblastoma** and **MM** treatment.	**↑** ROS: GSH pool depletion	**Phase I/II** study in MM (NCT00327249, completed in 2008). **Phase II** study for the treatment of aggressive lymphomas (NCT01314014, completed in 2014).
ATN-224	Under clinical trial.	**↑** ROS: SOD inhibitor	**Phase I/II** study of ATN-224 plus bortezomib in MM patients (NCT00352742, completed in 2008).
PX-12	Under clinical trial.	**↑** ROS: Thioredoxin system inhibitor	**Phase I** (NCT00736372, completed in 2009) and phase II studies (NCT00417287, completed in 2009) against solid tumours.
Phenethyl isothiocyanate (PEITC)	Under clinical trial.	**↑** ROS: Export GSH outside of the cell and inhibits GPx	**Phase I** study for preventing lung cancer (NCT00005883, completed). PEITC as support care in head and neck cancer patients (NCT03034603, recruiting).
GKT137831	Under clinical trial.	**↓**ROS: NOX1 and NOX4 inhibitor	**Phase II** study for the treatment of patient with type 2 diabetes (NCT02010242, completed in 2015).
Allopurinol	FDA-approved for **gout treatment** and as prophylaxis treatment to prevent chemotherapy-induced uric acid elevation.	**↓** ROS: XOD inhibitor	**Phase II** study of allopurinol in pediatric ALL treatment to improve 6-mercaptopurine treatment (NCT03022747, recruiting).

### Increasing oxidative stress contributes to the mechanism of action of anti-leukaemia chemotherapeutics

This section will discuss how some front-line anti-leukaemia chemotherapeutics alter the cellular redox balance, which could be important for their pharmacologic effect. A better understanding of this question would help to improve their therapeutic effect and overcome drug resistance.

AML still presents a great therapeutic challenge, of which the initial treatment cycle consists of arabinocytosine (a.k.a. cytarabine), a purine analogue that interferes with DNA replication, during 7 days, followed by anthracyclines antibiotics for 3 days. This is followed by additional chemotherapy or transplantation [137]. An increase in intracellular ROS has been found to be associated with the mechanism of action of cytarabine [138]. In this study the source of ROS was identified as coming from mitochondria. However, as reviewed elsewhere [139], anthracyclines could induce ROS formation via other mechanisms: (i) interacting with free iron and (ii) as by-products of their own metabolism by enzymes such as cytochrome P450 reductase, NADH dehydrogenase or XO and thioredoxin reductase (TrxR) [138]. NADPH oxidases are also implicated in anthracyclines-induced increase of ROS [140–142]. A recent study related the efficacy and toxicity of anthracyclines to NADPH oxidase polymorphisms [143]. The importance of ROS in the mechanism of action of anthracyclines was also highlighted by the fact that administration of antioxidants reduced their cytotoxicity [144]. In addition, it has been suggested that the enhancement of cellular antioxidant systems, such as SOD2 [145] or glutathione [146], could drive resistance to anthracyclines.

There is a significant number of novel compounds for AML treatment, whose feasibility is currently being tested in clinical trials [137]. Interestingly, the mechanism of action of some of these agents might be related to the alteration of redox homeostasis. Enzymes that control DNA epigenetic modifications, such as histone deacetylases (HDACs), are among their targets. HDACs inhibitors (HDACi), such as vorinostat, show anti-tumour capacity and increase ROS production. The use of antioxidants consistently reduces HDACi cytotoxicity [147], thus suggesting the importance of increased ROS production in the mechanism of action of HDACi. In addition, there is a study showing that AML cells resistant to HDACi exhibit higher antioxidant defences [148]. The aforementioned increase in ROS may be derived from mitochondria [147] and NADPH oxidases [148]. The mechanism of action of other epigenetic enzyme inhibitors currently being tested for AML treatment, such as Lysine-specific demethylase 1 (LSD1) [149] and Bromodomain and Extra-Terminal proteins (BET) inhibitors [150], could also be related to increases in ROS.

The cell cycle is another interesting molecular target in AML and cyclin-dependent kinases (CDKs) inhibitors are currently being studied in this respect [137]. Some work supports the fact that the antiproliferative effect of this type of inhibitors is linked to the induction of oxidative stress [151].

Leukaemic cells have a high concentration of ubiquitinated proteins [152]. Therefore, a number of proteasome inhibitors have been tested for anti-leukaemic activity. The first proteasome inhibitor approved by the FDA, bortezomib, has shown effectiveness against multiple myeloma (MM) [153], and it could also be used for AML [154] or CLL [155] treatment; induced oxidative stress is important for bortezomib cytotoxicity [156].

The treatment of acute promyelocytic leukaemia (APL) with the pro-differentiation agent *all-trans retinoic acid* (ATRA) is an effective strategy that would be interesting to treat other types of leukaemia [157]. In cases of relapsed APL, arsenic trioxide (As_2_O_3_, Trisenox) is a good drug choice, as it has been approved by the FDA against this disease in combination with ATRA and anthracyclines chemotherapy. Currently, As_2_O_3_ efficacy is being tested in newly diagnosed APL patients, with complete remission in 83–86% of patients and 3-year overall survival. As_2_O_3_ acts through different mechanisms such as stimulation of differentiation, induction of apoptosis, and NF-κB inhibition [158]. However, it seems that its main effect would be the induction of ROS accumulation. As_2_O_3_ can alter the cellular redox homeostasis at different levels: (i) by inducing electron leakage from the mitochondrial respiratory chain [159] and (ii) through the expression of the genes that code for the Nox2 complex-forming proteins [160]. As suggested by the irreversible inhibition of TrxR [161] and the depletion of peroxiredoxin III (Prx III) [162], As_2_O_3_ may also diminish the cellular antioxidant capacity, which would contribute to raise ROS levels. It is noteworthy that As_2_O_3_ cytotoxicity inversely correlates with the level of glutathione [163]. This is of key relevance, since the measurement of antioxidant defences in patient samples could help to predict their responsiveness to As_2_O_3_ or other pro-oxidant treatments, as previously reported for paclitaxel, a chemotherapeutic that also increases ROS [164]. This information could also help to improve the effect of As_2_O_3_, by simultaneously targeting various antioxidant cellular systems. In this regard, the reduction of glutathione has shown to increase the effectiveness of As_2_O_3_ [165]. Jeanne et al. have shown the importance of ROS production in the molecular mechanism of action of As_2_O_3_. The oxidative stress induced by As_2_O_3_ allows for PML/RARα dimerization through the formation of a disulfide bridge. Direct binding of As_2_O_3_ to PML/RARα dimers induces the formation of multimers and their association with the nuclear matrix in the so-called nuclear bodies (NBs), where eventually sumoylation and degradation of PML/RARα occurs [166].

A striking observation is that, despite differences between the mechanisms of action of all the previously described chemotherapeutics, they share oxidative stress as a mediator of their cytotoxic activity. This implies that in the past leukaemia was unintentionally targeted through redox mechanisms. The awareness of this fact may have important practical consequences, since it could help to improve therapeutic results, overcome resistances and reduce toxic side effects. Regarding the latter issue, the use of the tubulin inhibitor vincristine to treat ALL is associated with neurotoxicity [167]. The experimental evidence suggests that the cytotoxicity of vincristine and other tubulin inhibitors, such as paclitaxel, is related to the induction of oxidative stress [168]. Importantly, the concomitant use of antioxidant molecules reduced the toxic side effects of paclitaxel and other chemotherapeutics without compromising, or even improving, therapeutic results [169].

Given the complexity of leukaemia and the multiple factors contributing to its development, single-agent treatments are sometimes not satisfactorily effective; thus, combinatorial treatments are very common. The combination of two of the previously mentioned chemotherapeutics would ensure that two different biological processes relevant to leukaemic cells were targeted. Additionally, the combined treatment would increase oxidative stress (Table 1). Preclinical studies in leukaemic cell lines have shown a synergistic effect when combining HDACi with proteasome inhibitors, which is dependent on ROS production [170]. This result has led to two recently conducted phase II clinical trials in which the combination of bortezomib with vorinostat was tested in AML (NCT00818649) and ALL (NCT01312818). The combination of As_2_O_3_, bortezomib and L-ascorbate was recently evaluated in a clinical trial against relapsed or refractory MM with encouraging results [171]. Recent clinical trials have tested the combination of As_2_O_3_ with other compounds that also can increase ROS levels in AML patients. Examples are decitabine (NCT00671697, Phase I; NCT03381781, Phase II); citarabine and idarubicin (NCT00093483, Phase I); combination of altezumab with As_2_O_3_ and other chemotherapeutics (NCT00454480, Phase II/III).

### The ongoing search for novel chemotherapeutics that increase ROS levels

Given the oxidative stress “addiction” displayed by cancer cells, the promotion of their death through a ROS “over-dose” is becoming a popular idea. The realisation that many chemotherapeutics increase the oxidative stress has paved the way for the active search for novel compounds that can increase ROS levels, as recently reviewed [135]. Although some of these compounds may not be very effective as single agents, some reports suggest that they could potentiate the effect of traditional therapy and help to overcome resistance [172].

In the search for agents that alter cellular redox homeostasis, the mitochondrion should be one of the first targets considered. As reviewed elsewhere, the use of mitochondrial inhibitors is a suitable strategy for inducing oxidative stress with a therapeutic purpose in leukaemic cells [94]. Moreover, mitochondrion-targeting drugs might also activate the intrinsic apoptotic pathway. Interestingly, metformin, an antidiabetic drug, has been shown to inhibit mitochondrial ATP production and increase ROS [173]. This drug is currently under study in clinical trials for CLL (NCT01750567, phase II; NCT02948283, phase I), relapsed childhood ALL (NCT01324180, phase I), and relapsed/refractory AML (NCT01849276, phase I). Adaphostine, initially described as a TKi, increases ROS levels by inhibiting mitochondrial respiration [174], and can overcome resistance against imatinib in primary CML cells [175]. The safety of tigecycline, an antibiotic that inhibits mitochondrial biogenesis, has already been tested in AML patients (NCT01332786, Phase I) [176]. An in vitro study on the anti-leukaemic effect of tigecycline against cells from CML patients (NCT02883036) is also scheduled.

A recent report has shown the importance of limiting ROS production by cellular antioxidant defences for cancer initiation [177]. This work revealed that glutathione and thioredoxin are required for tumour initiation, and that inhibition of these antioxidant systems hinders tumour growth in a synergistic manner. This evidence strongly suggests that targeting the cellular antioxidant defences is an interesting strategy for fighting against tumour cells. In line with this, there is a growing list of preclinical studies testing the anti-tumour capacity of inhibitors for different antioxidant cellular systems, some of which will be discussed below.

The glutathione/glutathione peroxidase (GSH/GPx) system is a major regulator of redox homeostasis; therefore, its impairment may induce a severe oxidative stress in the cells. NOV-002, a complex of oxidised glutathione (GSSG) with cisplatin in a ratio of 1000:1, has shown effectiveness against non-small cell lung cancer (NSCLC) (NCT00347412*,* Phase III). Imexon acts by depleting glutathione levels [178]. Recent clinical trials have tested imexon in MM (NCT00327249, phase I/II) and aggressive lymphomas (NCT01314014, phase II). Similarly, glutathione depletion by buthionine sulfoximine (BSO) activates apoptosis in ALL cells [179] and increases As_2_O_3_ activity [180], suggesting that the use of this type of compounds to treat leukaemia could be an interesting option.

Different SOD inhibitors, such as ATN-224 [172] or 2-methoxyestradiol (2-ME, panzem) [181], have shown anti-leukaemic capacity in preclinical studies. Interestingly, a recent report has identified 2-ME as capable of targeting T-ALL pre-leukaemic stem cells without affecting normal HSCs in a high-throughput screening [182]. Recent clinical trials have tested the combination of ATN-224 and bortezomib in MM patients (NCT00352742, phase I/II), and 2-ME for targeting relapse or plateau phase myeloma (NCT00592579, phase II).

A conceptually opposing strategy could be proposed on the basis of some reports showing that SOD inhibits cancer cell growth. [183]. With this respect, the use of SOD mimetics in combination of other chemotherapeutics that increase ROS against cancer is being assessed [184]. These compounds may have antioxidant activity by decreasing superoxide levels, despite the fact that they can also increase the cellular level of H_2_O_2_. There is strong evidence supporting the idea that these mimetics would preferentially target highly dividing tumour cells, thus potentiating the effectiveness of other chemotherapeutics and reducing their toxic side effects as shown by a recent clinical trial (NCT00727922) [185].

Another body of research suggests that the thioredoxin redox system is an interesting drug target in cancer [186]. Several molecules interfere with this system, and some of them, such as PX-12 [187], gliotoxin [188], and chaetocin [189], have shown anti-leukaemic effects in preclinical studies. Recent clinical trials have tested PX-12 against solid tumours (NCT00736372, NCT00417287).

The heme oxygenase enzyme-1 (HO-1) catalyses the degradation of heme group to ferrous iron, biliverdin and carbon monoxide (CO). Under oxidative stress, HO-1 expression is induced by the NRF2 transcription factor as part of the cellular antioxidant defence response. HO-1 is upregulated in some leukaemic cells [190], which could be a compensatory mechanism to cope with oxidative stress [95]. It is noteworthy that HO-1 overexpression has been related to drug resistance [191]. There is experimental evidence showing that targeting HO-1 may be an interesting strategy to i) fight haematological malignancies, and ii) overcome the resistance to pro-oxidant drugs [190].

As previously mentioned, the activity of many transcription factors is inhibited by oxidation, where APE/Ref-1 catalyses the reduction of several of them [19]. APE/Ref-1 inhibition induces sensitivity to anti-tumour drugs [192]. This finding, as well as the development of several APE/Ref-1 inhibitors [193], suggests that this protein could be a promising therapeutic target in the treatment of leukaemia [192].

Another line of work in the search for pro-oxidant chemicals is testing the anti-tumour activity of natural derivatives, as some, such as parthenolide, triptolide and avocatin B, have shown effectiveness against AML cells [194]. Phenethyl isothiocyanate (PEITC), a compound present in cruciferous vegetables, has made a stellar apparition in the field of cancer therapeutics. There is a vast number of preclinical studies revealing the anti-tumour activity of PEITC, which is related to the induction of ROS [195]. Experimental evidence suggests that PEITC may increase sensitivity to chemotherapy in B-cell prolymphocytic leukaemia (B-PLL) patients [196], and overcome resistance in CLL [197] and CML [198]. Recent (NCT00005883, Phase I) and ongoing (NCT03034603) clinical trials will elucidate the potential benefit of using PEITC as a nutritional supplement against cancer.

Natural flavonoids can work either as ROS scavengers or pro-oxidants. Several preclinical reports showed that some natural flavonoids and their derivatives have pro-apoptotic and cytotoxic effects against different types of haematological malignancies. Interestingly, many of the anti-leukaemic effects described in the literature for these compounds are linked to an increase in oxidative stress [199].

### Reducing ROS levels as a chemotherapeutic strategy

It can be suggested that reducing ROS levels may restrain the development of these diseases, given the high degree of oxidative stress typical of haematological malignancies. In spite of this, and as discussed above, during the last decade most efforts have been made to kill tumour cells by a ROS overload. In this regard a common assumption is that an antioxidant-rich diet might reduce the incidence of leukaemia. However, the benefit of the antioxidants used during the oncologic treatment is a matter of debate. Some reports show that antioxidants can reduce the cytotoxicity of many chemotherapeutics. Taking this into account, it could be suggested that the use of antioxidants during an oncologic treatment would be unadvisable. However, there is also evidence showing that antioxidants could reduce the toxic side effects caused by pro-oxidant drugs [200]. Therefore, the use of antioxidants as adjuvants of oncologic treatments indeed requires further evaluation to discover the way in which therapeutic benefits can be attained [201].

An alternative to ROS-scavengers could involve the inhibition of the source(s) of ROS production, whose identification and further inhibition would be more effective than the use of antioxidants. As discussed above, NADPH oxidases could be one of the sources of ROS in leukaemic cells. In addition, the over-expression of some genes related to chemotherapeutic resistance, such as HO-1, is under NADPH oxidases control [202]. Considering this, NADPH oxidases appear to be suitable therapeutic targets in leukaemia. Preclinical data show that the inhibition of NADPH oxidases is an effective strategy to block the signalling cascades initiated by the BCR-ABL and FLT3-ITD oncokinases in CML and AML cells, respectively. This evidence supports the hypothesis that the aforementioned proteins induce NADPH oxidase-driven ROS production to maintain the signalling cascade fully active. Thus, the use of TKis and NADPH oxidase inhibitors presents a strong synergistic effect [81]. As discussed above, several oncogenes increase ROS production through NADPH oxidases, which turns these enzymes into desirable targets against leukaemia. However, the development of novel and more specific inhibitors against NADPH oxidases is still a challenge [203]. The safety and efficacy of two novel NADPH oxidases inhibitors (GKT136901 and GKT137831) have been tested in diabetic patients (NCT02010242). Once the suitability of these agents is demonstrated, their use could be extended to the treatment of certain types of leukaemia.

The inhibition of ROS production by XOD is another possibility for targeting leukaemic cells. Allopurinol, a XOD inhibitor currently prescribed to gout patients, has been considered since almost 50 years ago for the treatment of haematological malignancies [204]. In addition to modifying ROS levels, XOD is involved in the metabolism of several drugs. Recent reports suggest the utility of allopurinol to reduce chemotherapy toxicity in leukaemia patients [205]. An ongoing clinical trial is testing the feasibility of allopurinol to improve 6-mercaptopurine regimen in paediatric ALL treatment (NCT03022747, phase II).

IDHs mutations in AML patients induce an increase in ROS [206], and the use of IDH inhibitors can be seen as a promising strategy against AML [207]. Thus, some interesting questions to be addressed in future studies are whether these inhibitors have the ability to reduce the oxidative stress in leukaemic cells, and whether their anti-tumour activity may be increased by the use of other ROS-modifying agents.

### Practical implications for the haematopoietic transplant

Allogenic BM transplantation has been considered for decades as a good therapeutic option for different haematological diseases [208]. As a result, improving transplant outcome is of great importance. Although HSCs live in a low oxygen concentration niche, which limits the generation of ROS, the collection of donor HSCs is carried out under atmospheric conditions and could lead to the abrupt increase of ROS. Moreover, mobilization of HSCs in response to G-CSF is a common strategy for collecting human HSPCs. However, this mobilization occurs with an increase in ROS [209]. Given the importance of redox homeostasis to regulate HSCs quiescence and differentiation, it could be assumed that the limitation of ROS generation in transplantable HSCs would improve their reconstitution capacity.

There is a well-designed study conducted in an animal model showing that the collection of HSCs in hypoxic conditions significantly enhances transplant efficacy [210]. Furthermore, it has been recently described that the increase of ROS in HSCs correlates with a poor engraftment [211]. Therefore, it could be hypothesised that pharmacological mitigation of the accumulation of ROS could be also an interesting strategy to improve BM transplantation. In fact, a recent report shows that limiting mitochondrial oxidative stress with a MnSOD mimetic improves the function of HSCs in animal models of haematopoietic transplantation [212]. These findings encourage further studies for improving transplantation through modulation of ROS levels.

## Conclusions

The importance of ROS for the control of cellular signalling and gene expression is already a fully accepted concept. The level of ROS must be strictly regulated to control the biology of HSCs, suggesting the importance of redox signalling in haematopoiesis. Cancer cells, including leukaemic cells, live under oxidative stress and it is considered that an excessive level of ROS can contribute to proliferation and cell transformation. The potential sources of oxidative stress in leukaemic cells have been reviewed. The high level of ROS found in leukaemic cells certainly provides them with some advantages over healthy cells. However, this is a dangerous pathway, since, as already discussed, the alteration of ROS levels can jeopardise leukaemic cell proliferation and viability. The search for novel therapeutic strategies that either increase or reduce ROS levels represents an interesting approach to combat leukaemia. Moreover, there are different chemotherapeutic agents for treating leukaemia that are currently used in clinical practice whose activity relies on changes in the levels of ROS. This condition should be exploited in order to boost their activity.

Finally, given the importance of ROS for the control of HSCs biology, the modulation of ROS levels as a powerful strategy to improve the haematopoietic transplant is also proposed. Details regarding the involvement of ROS in haematopoiesis and leukaemogenesis are slowly beginning to be revealed. Future studies should be focused on defining the sources of ROS and their targets in haematopoiesis, which undoubtedly will help clinicians to improve the health of haematological patients.

## Abbreviations

2-HG: 2-hydroxyglutarate
2-ME: 2-methoxyestradiol
ALL: Acute lymphocytic leukaemia
AML: Acute myeloid leukaemia
APE1/Ref-1: AP endonuclease 1/redox factor 1
APL: Acute promyelocytic leukaemia
ARE: Antioxidant response elements
ATL: Adult T-cell leukaemia
*Atm*: *Ataxia–telangiectasia mutated gene*
ATRA: All-trans retinoic acid
BET: Bromodomain and extraterminal domain
BM: Bone marrow
BMSECs: BM sinusoidal endothelial cells
B-PLL: B-cell prolymphocytic leukaemia
BSO: Buthionine sulfoximine
CAR cells: CXCL-12-abundant reticular cells
CDKs: Cyclin-dependent kinases
CEL: Chronic eosinophilic leukaemia
Cell-SDF-1: Cell-derived factor-1, also named CXCL12
CGD: Chronic granulomatous disease
CLL: Chronic lymphocytic leukaemia
CML: Chronic myeloid leukaemia
CMML: Chronic myelomonocytic leukaemia
CO: Carbon monoxide
Cox-2: Cyclooxygenase-2
CXCL-12: CXC chemokine ligand 12
*CYP*: Cytochrome P-450 enzymes
FLT3: FMS-like tyrosine kinase-3
FLT3-ITD: FLT3-Internal Tandem Duplication
FOXO 4: Forkhead box protein O 4
FoxO: Forkhead O
G-CSF: Granulocyte colony-stimulating factor
GSSG: Oxidised glutathione
H_2_O_2_: Hydrogen peroxide
HDACi: HDACs inhibitors
HDACs: Histone deacetylases
HES: Hypereosinophilic syndrome
HIF-1: Hypoxia inducible factor 1
HO-1: Heme oxygenase enzyme-1
HSCs: Haematopoietic stem cells
HSPCs: Haematopoietic stem and progenitor cells
HTLV-1: Human T-cell leukaemia virus type 1
IDH1: Isocitrate dehydrogenase 1
JMML: Juvenile myelomonocytic leukaemia
LSC: Leukaemia stem cells
LSD-1: Lysine-specific demethylase 1
LT-HSCs: Long-term reconstitution HSCs
MAPK: Mitogen-activated protein kinase
MDS: Myelodysplastic syndromes
MM: Multiple myeloma
MPNs: Myeloproliferative neoplasm
MRC: Mitochondrial respiratory chain
mtDNA: mitochondrial DNA
mTOR: mammalian target of rapamycin
NBs: Nuclear bodies
NOS: NO synthases
Nox: NADPH oxidase
NRF2: Nuclear factor erythroid 2-related factor 2
NSCLC: Non-small cell lung cancer
O_2_^−^: Superoxide
OH^-^: Hydroxyl radical
PcG: Polycomb group
PEITC: Phenethyl isothiocyanate
PI3K: Phosphoinositide 3-kinase
Prx III: Peroxiredoxin III
PTPs: Protein tyrosine phosphatases
ROS: Reactive oxygen species
SOD: Superoxide dismutase
TKi: Tyrosine kinase inhibitors
TPO: Thrombopoietin
TrxR: Thioredoxin reductase
TSC: Tuberous sclerosis complex
XOD: Xanthine oxidoreductase
α-KG: α-ketoglutarate
